# Immune aging, dysmetabolism, and inflammation in neurological diseases

**DOI:** 10.3389/fnins.2015.00172

**Published:** 2015-06-03

**Authors:** Michela Deleidi, Madeline Jäggle, Graziella Rubino

**Affiliations:** ^1^Department of Neurodegenerative Diseases, German Center for Neurodegenerative Diseases (DZNE), Hertie Institute for Clinical Brain Research, University of TübingenTübingen, Germany; ^2^Department of Internal Medicine II, Center for Medical Research, University of TübingenTübingen, Germany

**Keywords:** brain-immune interactions, aging, immunosenescence, inflammation, metabolism, neurodegenerative diseases, neuropsychiatric diseases

## Abstract

As we age, the immune system undergoes a process of senescence accompanied by the increased production of proinflammatory cytokines, a chronic subclinical condition named as “inflammaging”. Emerging evidence from human and experimental models suggest that immune senescence also affects the central nervous system and promotes neuronal dysfunction, especially within susceptible neuronal populations. In this review we discuss the potential role of immune aging, inflammation and metabolic derangement in neurological diseases. The discovery of novel therapeutic strategies targeting age-linked inflammation may promote healthy brain aging and the treatment of neurodegenerative as well as neuropsychiatric disorders.

## Introduction

Aging is a complex phenomenon characterized by the progressive decline of physiological function and tissue homeostasis leading to increased vulnerability to degeneration and death. One of the most recognized effects of aging is the dysregulation of the immune system as a result of defects in both initiation and resolution of immune responses (*immunosenescence*) and chronic low-grade inflammation (*inflammaging*) (Franceschi et al., 2007; Montecino-Rodriguez et al., 2013). This chronic subclinical condition has been linked to an increased incidence of metabolic syndrome, atherosclerosis, cancer, and neurodegenerative diseases (Franceschi et al., 2007). Similarly, recent work suggests that microglia, the innate immune cells of the brain, undergo a process of senescence that may in turn contribute to the development of neurological diseases in elderly people (Wong, 2013). In this review we discuss recent advances on the link between immune aging and neurological disorders and we describe how the metabolic changes of the aging body, immunosenescence, and inflammation can exacerbate immunological contributions to neurodegenerative and neuropsychiatric diseases.

## Immunosenescence and inflammaging

As we age, the immune system undergoes a process of senescence characterized by a progressive decline in immune function associated with an increased frequency of infections and chronic diseases. Aging affects both the adaptive and the innate immune system (Larbi et al., 2011; Goronzy et al., 2012; Shaw et al., 2013). The most relevant changes are observed in the adaptive immune system; immunosenescence is characterized by a decrease of naïve T cells and a concomitant increase in memory cells, a progressive reduction of the TCR repertoire and decreased proliferation *in vitro* (Arnold et al., 2011; Frasca et al., 2011; Nikolich-Zugich et al., 2012; Fulop et al., 2013). With regard to innate immunity, studies in animal models and humans have shown age-associated alterations including myeloid skewing (Dykstra et al., 2011), impairment of neutrophil chemotaxis and effector function (Wenisch et al., 2000; Butcher et al., 2001; Fulop et al., 2004), defects in NK cells (Le Garff-Tavernier et al., 2010) and monocyte dysregulation (Hearps et al., 2012). Immunosenescence is accompanied by a low-grade chronic proinflammatory environment in multiple tissues characterized by increased production of proinflammatory cytokines, such as interleukin-6 (IL-6) and tumor necrosis factor alpha (TNF-α), acute-phase proteins, reactive oxygen species (ROS), and autoantibodies. This proinflammatory environment has been defined as “inflammaging” (Franceschi et al., 2000, 2007). Several mechanisms contribute to inflammaging, including the dysregulation of the adaptive vs. innate immunity (Franceschi et al., 2000) and cellular senescence. *Senescent cells* secrete a variety of proinflammatory cytokines, chemokines, growth factors and proteases collectively termed as senescence-associated secretory phenotype (SASP) (Freund et al., 2010; Chinta et al., 2014; Ovadya and Krizhanovsky, 2014). The SASP has beneficial effects, such as the reinforcement of the tumor-suppressing cell state (Acosta et al., 2008), prevention of fibrosis (Jun and Lau, 2010), and clearance of senescent and tumor cells (Xue et al., 2007). On the other hand, cellular senescence and the SASP drive the chronic inflammatory environment that is a major contributor to the development of aging-associated diseases. *Chronic infections* promote immunosenescence and inflammaging (Koch et al., 2007); cytomegalovirus (CMV) promotes age-like immune changes (Derhovanessian et al., 2011) and CMV reactivation has been associated with increased levels of IL-6 and TNF and premature mortality (Stowe et al., 2007; Roberts et al., 2010). However, further studies are needed to assess the link between CMV infection and inflammatory markers in the elderly (Bartlett et al., 2012). Other chronic infectious diseases such as HCV and HIV may also have a role in immunosenescence (Gruener et al., 2001; Zapata and Shaw, 2014). With effective antiretroviral therapy, the life expectancy of HIV patients has significantly improved and a link between chronic infection, immune dysregulation and age-related comorbidities has become evident (Deeks and Phillips, 2009; Lederman et al., 2013). Antigen-dependent clonal expansion of memory T cells as well as premature immune senescence have been shown in HIV patients (Appay et al., 2002, 2011). However, there is still debate as to whether HIV accelerates such immune aging (High et al., 2012). Interestingly, despite the increased life expectancy, the incidence of cognitive impairment in these patients remains high and mounting evidence suggests that persistent inflammation and immune dysregulation play a key role in HIV-associated cognitive disorders (Hong and Banks, 2015). Further corroborating evidence linking chronic infections, aging, and immunosenescence comes from experimental studies showing that lymphocytic choriomeningitis virus (LCMV) infection leads to a reduction of specific antiviral T cell responses in aged mice (Mekker et al., 2012).

Parasites such as Toxoplasma gondii (T. gondii) also contribute to immune dysregulation. Chronic infection with T. gondii is characterized by the presence of intraneuronal cysts that are controlled by the immune system (Suzuki et al., 2010). Growing evidence shows a link between chronic infection and CD8 T-cell dysfunction that in turn may promote the psychiatric disturbances often observed in these patients (Bhadra et al., 2011, 2013; Torrey et al., 2012). *Genetic predisposition* leading to an increased tendency toward uncontrolled inflammatory responses can also accelerate immunosenescence and inflammaging. With this respect, the Leiden Longevity Study (LLS) showed that individuals enriched for longevity genetic traits are less susceptible to CMV-associated immune alterations with aging (Derhovanessian et al., 2010). *Hormonal changes* such as the decreased production of estrogen or androgen also influence the secretion of cytokines (Maggio et al., 2006; Abu-Taha et al., 2009). Finally, *alterations of mitochondrial function* and *metabolic changes in the adipose tissue* contribute to immunosenescence and inflammaging.

## Brain cell senescence and neuro-inflammaging

Senescent and hyperactive microglia have been detected in the aged and diseased brain (Streit et al., 2004, 2009). Microglia are the innate immune cells of the central nervous system (CNS) and are involved in several physiological and pathological brain functions. They play essential roles in brain development and actively support neural circuitries and plasticity either through the removal of synaptic elements or the secretion of neurotransmitters and neurotrophic factors (Nimmerjahn et al., 2005; Wake et al., 2009; Kettenmann et al., 2011). During infections, brain injury or neurodegenerative diseases, resting microglia transform into an active state and release immune molecules including cytokines, ROS, and growth factors. Activated microglia exert beneficial functions such as the phagocytic clearance of pathogens and cellular debris. However, uncontrolled immune reactions contribute to neuronal dysfunction and death over time. During aging, rodent microglia undergo replicative senescence characterized by telomere shortening and loss of homogenous distribution throughout the brain (Flanary et al., 2007; Hefendehl et al., 2014). Experimental evidence suggest that microglial motility and paghocytic activity decrease with age leading to the formation of glial lipofuscin granules with subsequent accumulation of damaged mitochondria and oxidative stress (Brunk and Terman, 2002; Szweda et al., 2003; Xu et al., 2008; Damani et al., 2011; Hefendehl et al., 2014). In addition, histo-pathological studies show increased expression of interleukin-1α as well as CD11b/CR3 in humans suggesting that uncontrolled microglial activation occurs with normal brain aging (Sheng et al., 1998; Strohmeyer et al., 2002). Animal studies show increased microglial expression of MHC class II antigens and CD68 (Perry et al., 1993; Sheffield and Berman, 1998) as well as decreased arborization and abnormal cytoplasmic structures (Streit et al., 2004, 2009; Conde and Streit, 2006). Interestingly, these morphological changes precede the appearance of tau neuropathology in Alzheimer's disease (AD) patients suggesting that a loss of microglial function contributes to the onset of the disease (Streit et al., 2009).

The cellular and molecular mechanisms controlling brain immune senescence and neuro-inflammaging are still unknown. Several studies support the existence of a bidirectional communication between the peripheral immune system and the brain mainly through immune molecules, including cytokines, chemokines, acute phase proteins, and complement factors (Wrona, 2006). On the other hand, the aging brain is able to modulate the immune system and to promote the recruitment of immune cells from the periphery, especially lymphocytes, which contribute to immunosenescence and neuro-inflammaging (Gemechu and Bentivoglio, 2012). Thus, peripheral immunosenescence and inflammaging can modulate microglial phenotype and reactivity and drive low-grade brain inflammation (von Bernhardi et al., 2010). This hypothesis is supported by the observation that systemic low-grade inflammation is accompanied by an increase of cytokines in the brain and an imbalance between pro- and anti-inflammatory molecules (Ye and Johnson, 2001; Lukiw, 2004; Streit et al., 2004).

Systemic inflammation promotes the activation of brain immune cells in humans as well as in non-human primates (Brydon et al., 2008; Harrison et al., 2009; Hannestad et al., 2012) and can prime neurons and immune cells in the brain increasing the risk of developing neurological disorders (Deleidi and Isacson, 2012; Perry and Holmes, 2014). The molecular pathways that link low-grade systemic inflammation to brain aging and neuroinflammation remain unclear.

**Figure 1:**
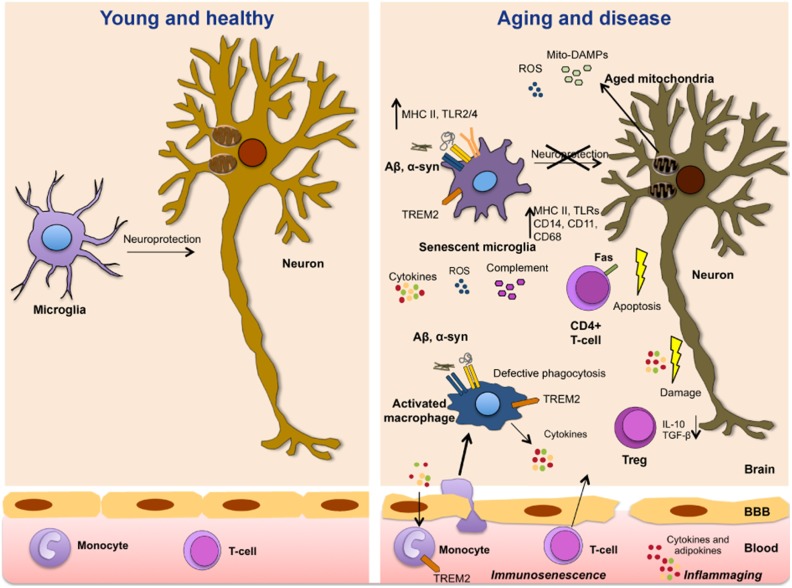
**Immune aging, dysmetabolism, and inflammation in neurological diseases **. The aging of the immune system is accompanied by a progressive senescence of immune cells (*immunosenescence*) and a chronic proinflammatory environment characterized by increased levels of cytokines and adipokines (*inflammaging*). These immune molecules modulate neuronal function and prime microglial cells and vulnerable neuronal populations. With aging, microglial cells also become senescent, lose their neuroprotective function and become more prone to abnormal inflammatory activation. Amyloid deposition and pathogenetic forms of α-synuclein activate microglia with the subsequent release of proinflammatory mediators. Aged dysfunctional mitochondria play a fundamental role in immunosenescence and age-related inflammation by the production of ROS and DAMPs that activate the NLRP3 inflammasome and immune cells triggering inflammatory reactions. In turn, inflammatory mediators aggravate mitochondrial dysfunction. High immune risk profile, infections and metabolic syndrome promote abnormal inflammatory reactions that contribute to disease onset and progression.

In summary, the aging of the peripheral immune system can promote or exacerbate microglial senescence and drive neuroinflammation (Figure 1). The individual genetic background as well as infections and environmental exposures over a lifetime could further exacerbate the dysregulation of immune responses, increasing the risk of neurological disorders.

## Aging, obesity, and neuroinflammation

Aging is characterized by important changes in the distribution of total and regional adipose tissue (Kuk et al., 2009). Overnutrition and obesity are associated with an increased risk of metabolic syndrome, a complex disorder characterized by hypertension, insulin resistance, glucose intolerance, and hyperlipidemia. Both hypertrophic fat cells and immune cells infiltrate the adipose tissue and secrete inflammatory molecules that can promote systemic low-grade inflammation. Adipocytes secrete more than 600 bioactive molecules, which are collectively termed as adipokines and include proinflammatory mediators, such as TNF-α, monocyte chemoattractant protein (MCP)-1, and IL-6, as well as anti-inflammatory molecules (Trayhurn and Wood, 2004). A link between obesity and inflammation has been initially hypothesized based on the observation of increased levels of IL-6 and C-reactive protein (CRP) in obese animals and humans (Yudkin et al., 1999; Festa et al., 2001; Moschen et al., 2011). Interestingly, concentrations of these cytokines decrease after weight loss (Ryan and Nicklas, 2004; Rao, 2012). Circulating adipokines have immune as well as neuronal functions and can influence brain inflammation. For example, leptin, one of the first adipokines identified, is a proinflammatory cytokine sharing structural similarities with IL-2, IL-6, IL-12, and granulocyte colony stimulating factor (Zhang et al., 1997). Leptin has important immune functions, especially related to T lymphocyte proliferation (Lord et al., 1998; Carbone et al., 2012). In the CNS, it also regulates appetite and energy balance (Friedman and Halaas, 1998) and contributes to development of sickness-type behavior during experimental inflammation (Pohl et al., 2014). Thus, excess adiposity drives low-grade systemic inflammation that increases the risk of developing insulin resistance, type 2 diabetes, cardiovascular disease, stroke, cancer, and neurodegeneration (Tchernof and Despres, 2013).

The link between obesity-induced inflammation and type 2 diabetes is further supported by the observation that the development of insulin resistance correlates with immune cell infiltration (Hotamisligil, 2006). Interestingly, obesity is also linked to mitochondrial dysfunction and endoplasmic reticulum (ER) stress (Arruda et al., 2014), which in turn leads to impaired glucose metabolism. In fact, the earliest hallmark of insulin resistance is the decreased expression of PPARγ coactivator 1-α (PGC1α), a positive regulator of mitochondrial biogenesis and respiration (Patti et al., 2003). Decreased expression of PGC1α has been linked to aging and chronic inflammation in humans and mice (Ghosh et al., 2011; Sczelecki et al., 2014).

In summary, fat accumulation and changes in fat redistribution with age lead to the dysregulation of adipokine secretion and metabolic changes linked to systemic and local inflammatory responses that, in turn, contribute to accelerated aging as well as cardiovascular and neurological disorders.

## Mitochondrial dysfunction in aging and inflammation

Because of their function in oxidative stress processes, mitochondria have a fundamental role in aging (Green et al., 2011). Altered mitochondrial function is central to several acute and chronic inflammatory diseases and increasing evidence show that mitochondria may contribute to immunosenescence and inflammaging via ROS production, calcium exchange, ATP and NF-κB activation. Mitochondrial dysfunction can be both the cause and the consequence of inflammation. Aged dysfunctional mitochondria are able to modulate innate immunity through redox-sensitive inflammatory signaling pathways or the direct activation of the NLRP3 inflammasome (through ROS, mtDNA, and ATP) (Kepp et al., 2011; Shimada et al., 2012). Mitochondria are an important source of damage-associated molecular patterns (mito-DAMPs) that activate Toll-like membrane receptors (TLRs) and cytoplasmic nucleotide-binding oligomerization domain (NOD)-like receptors (NLRs) (Krysko et al., 2011). Interestingly, DAMPs and mito-DAMPs directly activate antigen-presenting cells and other non-immune cells such as astrocytes (Mathew et al., 2012). On the other hand, inflammatory mediators, including inflammatory cytokines, ROS, and nitric oxide (NO), impair mitochondrial function by inducing the accumulation of mtDNA mutations and inhibiting mitochondrial respiratory chain and energy production (Lopez-Armada et al., 2013).

## Immune senescence and inflammation in neurological diseases

### Neuropsychiatric disorders

In recent years, the role of the immune system in the development of neuropsychiatric disorders has become more apparent. The immune system communicates with the brain and regulates behavior and many other critical neurological functions (Wilson et al., 2002). Thus, age-related immune dysregulation may be relevant to the pathophysiology of neuropsychiatric conditions. This hypothesis is further supported by the observation that psychiatric manifestations are common in autoimmune diseases including systemic lupus erythematosus, CNS vasculitis, Whipple's disease, Sjögren's syndrome, and Behçet's disease [reviewed in (Kayser and Dalmau, 2011)]. In addition, patients with acute isolated psychosis have been diagnosed with synaptic autoimmune encephalitis and antineuronal antibodies targeting synaptic proteins have been demonstrated (Kayser and Dalmau, 2011). Finally, it is well established that infections and inflammation during the perinatal period increase the risk of developing neurological and neuropsychiatric diseases in the adulthood (Hagberg et al., 2015). Infectious diseases can cause direct neuronal damage; in other circumstances, such as after LCMV infection or chronic T. gondii infection, neuronal dysfunction may also be linked to immune reactions. Chronic immune activation is a potential key contributor to disease pathology, even in the absence of overt cell death. It is also well established that viruses can alter neurotransmission and activate the kanyurine pathway causing chronic fatigue, cognitive impairment and mood disorders (Weissenborn et al., 2006). In particular, the alteration of the kanyurine pathway is linked to an increased risk for neurological and neuropsychiatric disorders (Schwarcz et al., 2012). It has also been hypothesized that viruses such as LCMV alter inhibitory circuits causing unbalanced excitatory neurotransmission and neuronal death via excitotoxicity (“Virus induced disinhibition and excitotoxicity”) (Pearce et al., 2000). Finally, long-term alterations in neurogenesis have been described in mice after LCMV infection (Sharma et al., 2002).

Several immune changes are associated with neuropsychiatric disorders; increased levels of inflammatory mediators have been reported in schizophrenia (Soderlund et al., 2009; Beumer et al., 2012a), depression (Dowlati et al., 2010) and autism spectrum disorders (ASD) (Vargas et al., 2005; Li et al., 2009). Altered monocytic as well as T- and B-cell function has been demonstrated in patients with schizophrenia and ASD (Ashwood et al., 2011; Beumer et al., 2012b; Braunschweig and van de Water, 2012; Jyonouchi et al., 2012; Muller et al., 2012). Further corroborating evidence comes from the observation that infections and chronic inflammatory diseases are linked to depression, anxiety as well as schizophrenia (Eaton et al., 2006; Loftis et al., 2008; Yolken and Torrey, 2008; Margaretten et al., 2011; Watkins and Treisman, 2012; Bhadra et al., 2013). Interestingly, many genes involved in the predisposition to schizophrenia are related to immune defense against invading pathogens, suggesting an involvement of immune dysregulation and infections in the pathogenesis of this disease (Carter, 2009; International Schizophrenia et al., 2009; Irish Schizophrenia Genomics C and the Wellcome Trust Case Control C, 2012). Neuropsychiatric disorders are also characterized by the activation of the brain innate immune system and increased density of activated microglia has been found in brains of schizophrenic and ASD patients (Vargas et al., 2005; Doorduin et al., 2009).

Aging, metabolic changes, obesity, and chronic stress modulate the communication between the immune system and the brain and may promote neuropsychiatric diseases. Neuropsychiatric disorders are indeed common in people with metabolic dysfunctions (Skilton et al., 2007). In these patients, inflammation is one major determinant of depressive symptoms (Capuron et al., 2008). Obesity is also a contributing factor; in obese women adiposity is associated with increased concentrations of inflammatory markers (IL-6, CRP, and adipokines) that correlate with depression and anxiety (Capuron et al., 2011). Interestingly, after surgery, weight loss correlates with reduced markers of inflammation and is associated with improvement of psychiatric symptoms (Capuron et al., 2011).

The brain, in turn, is able to regulate the immune system via neurotransmitter signals (Felten et al., 1987). As a consequence, psychological stress and depression are often associated with impaired immune function and increased risk of acute respiratory infections (Cohen et al., 1991, 2012). In addition, premature shortening of telomeres in leukocytes has been reported in subjects with chronic stress and depression as well as in mice exposed to stressful conditions (Epel et al., 2004; Simon et al., 2006; Kotrschal et al., 2007; Karabatsiakis et al., 2014). Stress responses pathways including the hypothalamic–pituitary–adrenal (HPA) axis and chronic immune stimulation, which are often associated with mood disorders, could be the cause of telomere shortening and accelerated aging observed in these patients (Glaser and Kiecolt-Glaser, 2005). The role of telomere shortening in other psychiatric disorders such as schizophrenia is still controversial and further studies are therefore warranted (Nieratschker et al., 2013; Kota et al., 2014).

### Alzheimer's disease and mild cognitive impairment

AD is the most common form of dementia in the elderly affecting more than 35 million people worldwide. In AD, the progressive neuronal loss in the medial temporal lobe and many other brain regions causes cognitive decline. The pathological hallmarks of AD are extracellular β-amyloid (Aβ) plaques, intracellular neurofibrillary tangles and gliosis consisting of activated microglia and astrocytes surrounding β-amyloid plaques (Joachim and Selkoe, 1992). The pathogenesis of the common sporadic forms of AD is still unknown. Neuroinflammation and microglial activation have been proposed as key players in the pathogenesis of the disease (Wyss-Coray, 2006). Inflammation is an early event in the amyloid pathology and precedes plaque deposition in experimental models of AD (Ferretti et al., 2012; Wright et al., 2013). Amyloid deposition leads to microglial activation (Meyer-Luehmann et al., 2008) and the production of proinflammatory mediators that contribute to disease pathogenesis. However, microglia also play a beneficial role in restricting senile plaque formation by clearing Aβ deposits (Simard et al., 2006) and secreting neuroprotective factors. It has been proposed that the loss of such neuroprotective function rather than microglial inflammatory activation contributes to AD. Microglial senescence and dysfunction have been described in AD (Streit et al., 2009). Oxidative stress-related mechanisms that precedes Aβ oligomerization can trigger microglia senescence and dystrophy (Sutherland et al., 2013). Senescent microglia upregulate immune receptors such as MHC II, CD68, CD14, CD11, and TLRs (Sheffield and Berman, 1998; Landreth and Reed-Geaghan, 2009; Hart et al., 2012) and become more prone to trigger inflammatory response to Aβ (Reed-Geaghan et al., 2009). Thus, loss and gain of microglial function are not mutually exclusive and can both play a role in disease pathogenesis and progression. Loss of function in peripheral monocytes and macrophages can also contribute to disease pathogenesis. It has been shown that, upon stimulation, monocytes from AD patients secrete more proinflammatory cytokines compared to monocytes from healthy subjects (Bonotis et al., 2008). In addition, phagocytosis of Aβ is defective in peripheral macrophages from AD patients and this may elicit a compensatory response of the adaptive immune system (Fiala et al., 2005). AD-linked genetic mutations and metabolic risk factors such as obesity, lipid dysmetabolism, and chronic inflammation can trigger immune cell dysfunction and promote cognitive decline. Interestingly, two recent GWAS studies have identified mutations in *Trem2* (triggering receptor expressed on myeloid cells 2) as risk factors for AD (Guerreiro et al., 2013; Jonsson et al., 2013). TREM2 is an innate immune receptor expressed on the cell surface of microglia, macrophages, osteoclasts and immature dendritic cells (Colonna, 2003). It regulates the phagocytic ability of myeloid cells and has anti-inflammatory properties by inhibiting cytokine secretion (Takahashi et al., 2005; Turnbull et al., 2006). Apolipoprotein E allele ε4 (APOE^*^ε4), the best-established genetic risk factor for sporadic AD, can also influence the degree of microglial activation in response to Aβ deposition (Barger and Harmon, 1997; Rodriguez et al., 2014). Associations have been reported between APOE^*^ε4 and clinical outcomes in infectious diseases further supporting a link between the patient genetic profile and the degree of inflammatory responses (Gerard et al., 2005; Itzhaki and Wozniak, 2006).

Metabolic syndrome has been associated with impaired cognitive function and increased risk of AD (Razay et al., 2007). Patients with diabetes mellitus have an increased risk of developing AD (Arvanitakis et al., 2004). Several studies show a negative correlation between body mass index (BMI) and cognitive performance (Elias et al., 2005; Jeong et al., 2005; Hassing et al., 2010). The mechanisms by which obesity influences risk of AD are still unclear. Obesity may contribute to disease onset by promoting low-grade systemic inflammation (Odegaard and Chawla, 2013; Erion et al., 2014), the disruption of the integrity of the blood brain barrier (Gustafson et al., 2007), and altered lipid metabolism (Hottman et al., 2014). Associations between systemic inflammatory markers and cognitive decline have been described with conflicting results and further studies are warranted (Engelhart et al., 2004; Tan et al., 2007; Sundelof et al., 2009; Singh-Manoux et al., 2014).

In summary, aged-related metabolic changes, immunosenescence and inflammaging can induce loss of microglial function and promote neuroinflammation and Aβ accumulation over time (Figure 1).

### Amyotrophic lateral sclerosis

Amyotrophic lateral sclerosis (ALS) is an adult-onset neurodegenerative disease that affects motor neurons in the brain and spinal cord leading to progressive paralysis and death within 2 to 5 years after onset of symptoms. The etiology of ALS remains largely unknown. Most cases are sporadic, whereas approximately 5–10% of the cases are caused by an autosomal dominant mutation. The pathological hallmarks of ALS are the presence of cytoplasmic ubiquitinated protein inclusions in degenerating motor neurons and intense inflammatory reactions characterized by microglial/astroglial activation and infiltration of peripheral T cells (Pasinelli and Brown, 2006). Thus, both innate and adaptive immune responses are involved in the pathogenesis of ALS (McGeer and McGeer, 2002; Henkel et al., 2004). Experimental and clinical evidence support the hypothesis that microglia activation occurs at early stages of disease (Turner et al., 2004). In experimental ALS, the microglial phenotype changes with disease progression, with a shift from the M2 phenotype in the early disease phase to M1 phenotype at later stages, suggesting that the decreased function of neuroprotective microglia correlates with disease progression (Beers et al., 2011; Liao et al., 2012). Moreover, the degeneration of peripheral motor axons is preceded by the recruitment and activation of macrophages (Chiu et al., 2009). T cells also play a role in ALS as suggested by the presence of CD4+ and CD8+ T lymphocyte infiltrates surrounding degenerating motor neurons (Troost et al., 1989; Engelhardt et al., 1993). Recent studies have shown that regulatory T lymphocytes (Tregs) and cytotoxic T lymphocytes (Teffs) have distinct roles in the pathogenesis and progression of ALS (Beers et al., 2011). Increased numbers of endogenous Tregs and M2 microglia predominate at early stages of disease, whereas Th1 lymphocytes and M1 microglia are more abundant during the rapidly progressing phase (Beers et al., 2011; Liao et al., 2012). These data suggest that inflammation plays a neuroprotective role during the initial stages, whereas immune dysfunction correlates with the progression of the disease at later stages. With regard to ALS, it is still unclear whether immunosenescence, inflammaging, and dysmetabolism affect the disease risk. ALS is associated with metabolic alterations, including include weight loss, hypermetabolism, and hyperlipidemia (Dupuis et al., 2011), and dyslipidemia has been described as a protective factor in ALS (Dupuis et al., 2008; Sutedja et al., 2011). However, further research is needed to clarify the relationship between aged-associated metabolic derangements, inflammation and sporadic ALS.

### Parkinson's disease

PD is the second most common neurodegenerative disorder affecting over 4 million people worldwide. In PD, the interaction between aging, individual genetic vulnerability, and environmental factors leads to the death of dopaminergic (DA) neurons in the substantia nigra (SN) pars compacta and other vulnerable neuronal populations. Interestingly, the dysfunction of the autonomic nervous system (ANS) and brainstem pathology precedes midbrain DA cell loss and motor symptoms (Braak et al., 2006). Even though inflammation has been associated with the pathogenesis of PD, it is still under debate whether this is a cause or consequence to neuronal death. Several lines of evidence support a role for both the innate and the adaptive immune system in disease pathogenesis. These include the increased levels of proinflammatory cytokines observed in PD patients (Dobbs et al., 1999) and the association of the disease risk with certain *HLA* genetic variants (Hamza et al., 2010). In PD, both native and pathogenic forms of α-synuclein activate microglial TLRs (TLR2 and TLR4) triggering the release of proinflammatory cytokines and ROS (Beraud et al., 2011; Stefanova et al., 2011; Codolo et al., 2013; Fellner et al., 2013). Interestingly, nitrated forms of α-synuclein exacerbate peripheral adaptive immune responses (Benner et al., 2008).

Metabolic syndrome has been proposed as a risk factor for PD. Increased BMI and high cholesterol are potential risk factors for PD (Hu et al., 2006, 2008). However, studies have yielded inconsistent findings and further investigations are needed to elucidate this association (Palacios et al., 2011). Diabetes increases PD risk in prospective studies and has been associated with severity of disease (Xu et al., 2011; Cereda et al., 2012). It has been suggested that common dysregulated pathways converging on mitochondrial dysfunction, ER stress, and inflammation as well as glucose and lipid metabolism, link diabetes, and PD (Santiago and Potashkin, 2013). Interestingly, high calorie and high fat diet exacerbates autonomic abnormalities in α-synuclein mutant mice suggesting a link between metabolism and disease pathology (Griffioen et al., 2013; Rotermund et al., 2014). It has been proposed that energy intake influences the vulnerability of neurons during aging by modulating the production of neurotrophic factors and inflammation (Maswood et al., 2004; Youm et al., 2015).

The role of the systemic immune system in PD is still unclear. It has been shown that peripheral monocytes from PD patients have a proinflammatory phenotype and impaired phagocytic function compared to controls (Grozdanov et al., 2014). In addition, T-lymphocytes, but not B-lymphocytes, are present in the SN of MPTP-intoxicated mice and PD patients (Brochard et al., 2009). Abnormalities in peripheral T cells, including decrease in the number of CD4(+) T cell subsets and Treg dysfunction have also been demonstrated in PD patients (Calopa et al., 2010; Saunders et al., 2012). These data support the existence of a peripheral immune dysfunction in PD and are complemented by the association of viral infections with disease onset. Viral infections and inflammatory reactions are a possible environmental trigger for PD (Deleidi and Isacson, 2012). A viral etiology for PD is based on epidemiological studies showing a high incidence of severe progressive parkinsonism in people developing encephalopathy after the 1918–1919 influenza outbreak (Ravenholt and Foege, 1982). In addition, parkinsonism has also been described in patients infected with other viruses such as Japanese encephalitis virus, Epstein-Barr virus, Coxsackie, St. Louis, West Nile and HIV viruses (Jang et al., 2009). Even though viral parkinsonism is extremely rare, an emerging hypothesis is that viral infections cause inflammatory reactions that prime vulnerable neurons to degenerate in response to other cellular insults over time (Deleidi et al., 2010). Finally, as many PD-linked genetic mutations play a role in the regulation of the immune system (Deleidi and Gasser, 2013), it is likely that genetic vulnerability predisposes to the development of midbrain DA neurodegeneration via inflammatory mechanisms.

In summary, more work is required to determine the role of age-linked inflammatory changes and dysmetabolism in the pathogenesis of PD. Nevertheless, emerging evidence suggest that the interplay between aging and genetic vulnerability results in increased susceptibility to infections or abnormal inflammatory reactions that overtime predispose to PD. A fundamental question to address is whether metabolic and inflammatory changes are strong predictors of neurodegeneration in PD patients. Understanding the molecular mechanisms regulating such responses and their link with aging and metabolism may provide potential opportunities for therapeutic targeting in PD.

### Multiple sclerosis

Multiple sclerosis (MS) is a chronic inflammatory demyelinating disease of the CNS in which the interaction between environmental exposures and genetic predisposition leads to persistent inflammation, oligodendrocyte death, and loss of the myelin sheath. Even though the sequence of events that initiate the disease remains largely unknown, MS is generally believed to be an immune-mediated disorder that occurs in genetically susceptible people. Diverse disease processes, including autoimmunity, viral infections, and metabolic changes may lead to the formation of inflammatory demyelinated plaques (Hedstrom et al., 2014; Belbasis et al., 2015). An accelerated aging could also play a role in the development of autoimmune diseases such as MS. Premature immune aging has been described in MS patients (Thewissen et al., 2005, 2007). It has been hypothesized that both genetic and environmental factors such as viral infections accelerate immunosenescence in these patients and contribute to disease pathogenesis.

## Therapeutic considerations

Potential therapeutic strategies targeting the age-associated inflammatory immune phenotype include anti-inflammatory drugs and lifestyle interventions such as diet, exercise and nutritional modifications. Common *anti-inflammatory drugs*, vaccines against CMV and HIV and novel strategies, either small molecules or monoclonal antibodies, targeting inflammatory pathways (e.g., IL1-β, TNFα, IL-6, mTOR), could be used in mono- or combination therapy. The association between nonsteroidal anti-inflammatory drug (NSAID) treatment and lower incidence of AD and PD is still controversial (in t' Veld et al., 2001; Chen et al., 2003; Wahner et al., 2007; Gao et al., 2011; Rees et al., 2011; Cote et al., 2012; Alzheimer's Disease Anti-inflammatory Prevention Trial Research, 2013). Epidemiological studies suggest that the risk reduction with NSAIDs decreases with age (Zandi et al., 2002) and may be affected by individual genetic traits such as the APOE genotype (Szekely et al., 2008). Further studies are therefore warranted to assess the role of anti-inflammatory drugs and identify patient subgroups that could benefit from such interventions. With respect to neuropsychiatric disorders, it is noteworthy that many antipsychotics and antidepressants decrease the levels of pro-inflammatory cytokines and inhibit immune-inflammatory pathways in humans and experimental models of inflammation (Miller et al., 2011; Walker, 2013). Interestingly, Tyring and colleagues showed that Etanercept, an anti-TNF-α molecule, improves fatigue and depression symptoms in patients with psoriasis (Tyring et al., 2006). Therapeutic strategies targeting *cellular senescence* and *SASP-induced inflammation* may be beneficial to dampen inflammaging and delay the onset and progression of neurological diseases (Wang et al., 2010; Tchkonia et al., 2013). Ideally, these drugs should delay the accumulation of senescent cells or promote their clearance, without interfering with anti-oncogenic pathways. With this respect, *inducers of heat shock response* (e.g., heat shock, physical exercise, and diet) can efficiently inhibit the SASP and reduce obesity-dependent chronic inflammation (Newsholme and de Bittencourt, 2014).

The *NLRP3 inflammasome* is another potential therapeutic target. The NLRP3 inflammasome is an immune sensor that could link systemic and brain aged-related inflammation (Youm et al., 2013). Interestingly, Youm et al. showed that caloric restriction in animal models reduces chronic inflammation with a mechanism involving the inhibition of NLRP3 inflammasome and reduced levels of IL-1β and IL-18 in human monocytes by the ketone metabolite β-hydroxybutyrate (Youm et al., 2015). Thus, dietary or pharmacological approaches should be exploited to treat NLRP3-mediated inflammatory diseases.

*Calorie restriction* improves health and survival in several species including non-human primates (Colman et al., 2014; Fontana and Partridge, 2015). Even though it is still an open question whether it extends lifespan in humans, several beneficial effects of calorie restriction have been shown including the improvement of cognitive function in elderly people (Witte et al., 2009) and a shift of the transcriptional profile toward a younger state in muscle cells (Mercken et al., 2013). Further understanding of the molecular mechanisms underlying the beneficial effects of calorie restriction in humans will help devise novel drugs and dietary supplements that can prevent or delay aging-related diseases.

*Nutritional supplements* such as natural anti-inflammatory molecules can dampen the inflammatory environment and promote healthy aging. For example, omega-3 essential fatty acids decrease the levels of IL-1, IL-6, TNF, and CRP (Simopoulos, 2002) and improve cognitive function in aged mice (Cutuli et al., 2014).

Regular and moderate *physical activity* is the most effective and affordable prevention strategy that reduces aged- and obesity-linked inflammation through the increased release of immunomodulant molecules (Cotman et al., 2007; Handschin and Spiegelman, 2008). Exercise improves cognitive, cardiac and immune function and reduces risk factors such as diabetes, hypertension, and cardiovascular diseases (Phillips et al., 2014). Interestingly, exercise also improves mitochondrial biogenesis and respiration by increasing the expression of PGC1α that, in turn, may have anti-inflammatory properties (Handschin and Spiegelman, 2008). Drugs or nutritional supplements that *enhance mitochondrial function* represent a potential therapeutic strategy for diseases associated with chronic low-grade inflammation.

Finally, it is important to remark that in current drug trials, inclusion criteria are usually based on clinical symptoms, therefore failing to take into account the underlying heterogeneity of the molecular pathogenesis and genetic risk patterns of complex neurological diseases. Thus, identifying the correlation between genomic risk profiles and the dysregulation of specific molecular inflammatory pathways will allow the identification of novel targets for therapeutic intervention and the stratification of patients toward personalized medicine.

## Conclusions

Aging predisposes to disease via several mechanisms that in part converge on inflammatory pathways. As we age, the immune system undergoes a process of senescence accompanied by the increased production of inflammatory cytokines. Given the bidirectional communication between the peripheral immune system and the brain, it is not surprising that systemic inflammation and metabolic changes interfere with brain immunological processes and neuronal function. Immune challenges such as systemic infections, age-related immunological and metabolic changes can alter this communication and disrupt brain plasticity. Under these circumstances, immune molecules in the periphery signal to neurons and glial cells via humoral routes or the sympathetic nervous system. Numerous studies have now provided evidence that increased levels of inflammatory molecules impair behavioral and cognitive performances (Lynch, 2010). Hence, this strengthens the hypothesis that the age-associated dysregulation of immune function and systemic as well as brain inflammaging contribute to increased risk of cognitive dysfunction and neurodegenerative diseases.

In summary, the interplay between aging, genetic predisposition, and environmental exposures initiate systemic and local metabolic changes as well as inflammatory reactions that predispose to neuropsychiatric and neurodegenerative diseases (Figure 1). We would further argue that targeting age-associated inflammation, especially in selected patients with high immune risk profiles, can be an advantageous strategy to prevent or delay the onset of these diseases. Further understanding the cellular and molecular mechanisms that control immunosenescence and inflammaging both in the periphery and in the brain will help unravel how the immune and nervous system communicate and devise novel drugs to promote healthy aging.

### Conflict of interest statement

The authors declare that the research was conducted in the absence of any commercial or financial relationships that could be construed as a potential conflict of interest.
